# Predicting transmission blocking potential of anti-malarial compounds in the Mosquito Feeding Assay using *Plasmodium falciparum* Male Gamete Inhibition Assay

**DOI:** 10.1038/s41598-018-26125-w

**Published:** 2018-05-17

**Authors:** Gonzalo Colmenarejo, Sonia Lozano, Carolina González-Cortés, David Calvo, Juliana Sanchez-Garcia, Jesús-Luís Presa Matilla, Didier Leroy, Janneth Rodrigues

**Affiliations:** 1 Diseases of the Developing World (DDW), GlaxoSmithKline, Severo Ochoa 2, Tres Cantos, 28760 Madrid, Spain; 2 In vivo Science & Delivery (IVSD), GlaxoSmithKline, Severo Ochoa 2, Tres Cantos, 28760 Madrid, Spain; 3 Medicines for Malaria Venture, Route de Pré-Bois 20, 1215 Geneva 15, Switzerland; 4 Present Address: Biostatistics and Bioinformatics Unit, IMDEA Food Institute, CEI UAM+CSIC, Ctra Cantoblanco 8, 28049 Madrid, Spain; 5 Present Address: Complejo Asistencial Universitario de León. Altos de Nava s/n, 24071 León, Spain

## Abstract

*Plasmodium falciparum* Standard Membrane Feeding Assay (*Pf*SMFA) is the current gold standard mosquito based confirmatory transmission blocking (TrB) assay for human malaria. However, owing to its complexity only selected gametocytocidal molecules are progressed into SMFA. Predictive tools for evaluation of TrB behavior of compounds in SMFA would be extremely beneficial, but lack of substantially large data sets from many mosquito feeds preempts the ability to perform correlations between outcomes from *in vitro* assays and SMFA. Here, a total of 44 different anti-malarial compounds were screened for inhibitory effect on male gamete formation in exflagellation inhibition assay (EIA) and the same drug-treated parasites were fed to mosquitoes in SMFA. Regression analysis was performed between outcomes of the two assays and regression models were applied to a randomly selected validation set of four compounds indicating no overfitting and good predictive power. In addition, the pIC50 for 11 different compounds obtained in the EIA was also correlated with pIC50’s in SMFA. Resulting regression models provided pIC50 predictions in SMFA with reasonably good accuracy thereby demonstrating the use of a simple *in vitro* assay to predict TrB of molecules in a complex mosquito based assay.

## Introduction

Malaria, the deadly infectious disease caused by the apicomplexan *Plasmodium* parasite and transmitted to humans by the *Anopheline* mosquito vector, has resulted in an estimated 216 million cases and 445,000 deaths globally in 2016, affecting the poorest of countries, mostly in the tropical and sub-tropical regions and the most vulnerable people (pregnant women, infants and children under the age of five years)^1^. Ongoing global efforts for malaria eradication have resulted in a significant reduction in new malaria cases with a 29% decrease in malaria deaths between 2010 and 2015, due in large to increased accessibility to key intervention tools like insecticide-treated nets (ITNs), Indoor Residual Spraying (IRS) and artemisinin-based combination therapy (ACTS) in sub-Saharan African countries with high malaria burden^2^. But, in 2016, 5 million more malaria cases were estimated to have occurred globally compared to 2015^1^. Additionally, the emergence and spread of resistance of *Plasmodium* to anti-malarial drugs is a major impediment towards global eradication efforts, and there is an urgent need to develop novel medicines that not only treat symptomatic malaria and cure the patient but which can interfere with transmission by the mosquito vector. Preventing malaria transmission is key to achieving goals of the malaria eradication agenda (MalERA)^3,4^, leading to renewed prioritization for discovery and development of molecules having target candidate profiles (TCPs) dedicated towards activity against parasite sexual stages^5^. *Plasmodium falciparum* is the deadliest of the 5 different species of *Plasmodium* that can cause human malaria, and transmissible sexual stages or gametocytes of this species comprise five distinct morphological stages which mature slowly over a period of 8–10 days. Mature male and female Stage V’s circulate in blood unlike the other sexual stages which are sequestered in tissues. Drugs and vaccines that kill or functionally inactivate Stage V gametocytes can disrupt this cycle. In recent years, large diverse chemical libraries have been screened for activity against mature gametocytes using several high throughput (HT) *in vitro* assays with different readouts however, gametocytocidal hit rates were low, mainly due to the slow rate of metabolic activity in mature gametocytes which renders them more refractive compared to asexual forms of the parasite^5,6^. Although HT gametocytocidal assays are helpful in providing hits, these molecules may not always be effective in reducing transmission to mosquitoes. Male gametocytes have been shown to be more sensitive to anti-malarial drugs compared to females^7^, and recently a single assay, the *P*. *falciparum* dual gamete forming assay (*Pf*DGFA) has been developed based on functional viability of male and female gametocytes in a HT format, enabling selection of compounds which can prevent mosquito infectivity^8^.

In screening cascades for either gametocytocidal drugs or vaccine candidates, the final confirmatory assay for measuring transmission blocking is the mosquito based SMFA^9–11^. Although the translatability of SMFA data to the clinic remains to be established, this assay currently continues to be the gold standard highest biological content assay to study transmission reduction^5,12^. The assay uses an *in-vivo* biological system, the mosquito vector, as a parasite readout interface to determine the Transmission Blocking Potential (TrBP) of drugs or vaccines. Both, mosquito-infective *Plasmodium* cultures and adult mosquitoes are essential for SMFA and the subsequent need for micro-dissections of individual mosquitoes for midguts combined with microscopic enumeration of oocysts, make it a labor-intensive, low throughput assay. Progression of compounds into this complex assay therefore requires stringent pre-selection criteria. Very few laboratories have access to insectaries, added to which, assay complexity and cost make it difficult to advance all hits from *in vitro* screening campaigns for evaluation into SMFA. This dearth of substantial information precludes the ability to predict the efficacy of *in vitro* assays in the mosquito. Lack of such predictive tools therefore hinders the ability to estimate TrBP of molecules which could have the highest degree of success in the mosquito. With the aim of discovering medicines with TrBP, GlaxoSmithkline (GSK), in collaboration with Medicines for Malaria Venture (MMV), set up SMFA within its facilities at the Tres Cantos Medicines Development Campus in Spain. To date, we have screened more than 50 compounds from both GSK and MMV partners for TrB activity in mosquitoes, and these include molecules in clinical and pre-clinical development, leads and late leads. Prior to performing mosquito feeds, we ensured that *in vitro* cultured gametocytes were viable by their ability to exflagellate; a drop in temperature of culture triggers a time dependent release of up to 8 flagellated male gametes from a single male gametocyte, resulting from three rounds of DNA replication^13^. Viability of both, drug treated and untreated (DMSO-controls) mature gametocyte *in vitro* cultures was determined using exflagellation as a read-out and these cultures subsequently fed to mosquitoes. Exflagellation Inhibition Assay (EIA)^14^ measures the ability of a compound to inhibit the number of exflagellation centers/field in drug-treated gametocyte cultures (either at a single concentration or a given set of concentrations) compared to DMSO-treated controls.

The percentage exflagellation inhibition (EI) obtained from the EIA was calculated using the equation:

1 $$EI=\,\frac{{E}_{C-}{E}_{T}}{{E}_{C}}\ast 100$$

where E_C_ and E_T_ are the number of exflagellation centers per field in the control and in the compound-treated sample, respectively.

The same treated and un-treated gametocyte cultures which were used in EIA were fed to mosquitoes in SMFA and the output was measured by enumeration of midgut oocysts at seven days post-feeding, by considering two different infection outcomes; (i) total number of *P*. *falciparum* oocysts per mosquito midgut (oocyst intensity) and its mean was estimated, and (ii) the total number of mosquitoes infected or prevalence of infection.

The percentage reduction of mean oocyst intensity (OR) was defined as,

2 $$OR=\,\frac{{O}_{C-}{O}_{T}}{{O}_{C}}\ast 100$$

where *O*_*C*_ and *O*_*T*_ were the mean oocyst intensity in the control and the compound-treated sample, respectively.

The percentage reduction in the prevalence of infection or block in transmission (BIT), was defined as,

3 $$BIT=\,\frac{{P}_{C-}{P}_{T}}{{P}_{C}}\ast 100$$

where *P*_*C*_ and *P*_*T*_ are the prevalence’s of infection in the control and the compound-treated sample, respectively.

In this study, we used data from a total of 44 different compounds (Table 1), either from GSK or MMV partners. All compounds were selected for progression into SMFA based on pre-established activity (in the nanomolar range) in at least one of several reported gametocyte stage V assays; compounds from GSK were screend in the ATP-based gametocytocidal assay^15^ and/or female gamete activation assay (FGAA)^16^. Compounds from MMV partners were selected based on actvity in at least one of the following assays; dual readout GFP-Mitotracker Red assay^17^, Saponin-lysis Sexual Stage Assay (SaLSSA)^18^, ATP assay^15^, FGAA^16^ and *Pf*DGFA^8^. Using EIA and SMFA data from these 44 different compounds, corresponding to a total of 148 sample points, we performed regression analysis between the EI and OR and EI and BIT. Compounds were tested at different concentrations ranging from 0,001 µM to 10 µM involving a total of 16,629 mosquitoes. Regression was used to derive models which were subsequently used for prediction and validation of transmission blocking behavior in the mosquito using a randomly selected set of compounds. Based on these results we further proposed a simple pathway for progression of molecules in SMFA thereby enabling the efficient use of mosquitoes and assay slots for screening molecules with TrB activity.

**Table 1:** List of 44 different compounds used.

Compound Number^@^	Compound ID	Compound Description	Concentrations used (µM)
1	DHA	Dihydroartemisinin (SIGMA)	0.001–1
2	Methylene Blue	Methylene Blue (SIGMA)	1
3	GSK^*^	NA	0.05–5
4	GSK^*^	NA	0.05–5
5	MMV000073	NITD609	0.005–0.5
6	MMV643121	DDD107498	0.0005–0.5
7	MMV000019	OZ439	0.001–10
8	MMV390048	PCUCTAN-126	0.005–0.5
9	MMV000100	ACT451840	0.005–0.5
10	MMV^*^	NA	0.01–10
11	MMV^*^	NA	1.0–10
12	MMV000147	P218	0.01–10
13	MMV390482	SJ557733	0.01–10
14	MMV^*^	NA	10
15	MMV^*^	NA	10
16	MMV^*^	NA	5
17	DDD1005283	Halofuginone	0.001–0.1
18	GSK^*^	NA	0.05–5
19	GSK^*^	NA	0.1–10
20	GSK^*^	NA	0.004
21	TCMDC-123475	TCAMS	10
22	TCMDC-125849	TCAMS	2
23	TCMDC-125487	TCAMS	5
24	TCMDC-125133	TCAMS	2.5
25	TCMDC-137453	TCAMS	5
26	TCMDC-141070	TCAMS	2
27	TCMDC-124559	TCAMS	1
28	TCMDC-125345	TCAMS	1
29	TCMDC-141154	TCAMS	1
30	TCMDC-141698	TCAMS	1
31	TCMDC-123767	TCAMS	1
32	GSK^*^	NA	1
33	GSK^*^	NA	2
34	GSK^*^	NA	0.001–1
35	MMV^*^	NA	0.001–5
36	MMV^*^	NA	0.001–1
37	MMV^*^	NA	0.03–1.5
38	MMV000039	Artemisone	0.03–1
39	MMV^*^	NA	0.1–1
40	GSK^*^	NA	1
41	MMV000080	Artemiside	0.003–1
42	MMV000020	OZ277	0.01–1
43	GSK^*^	NA	0.025–0.1
44	GSK^*^	NA	0.1

EIA and SMFA data was generated from a total of 44 different compounds from GSK and from MMV partners, belonging to different chemical classes and were either leads, late leads, pre-clinical candidates or in clinical stages of development.

Compound Number^@^ = Refers to Number used in entire Manuscript.

GSK^*^ = Compound ID not revealed.

MMV^*^ = Compound ID not revealed.

TCAMS = Tres Cantos Antimalarial Set.

NA = Not Applicable.

## Results

### Relationship between Exflagellation Inhibition (EI) and Oocyst Reduction (OR)

**Figure 1:**
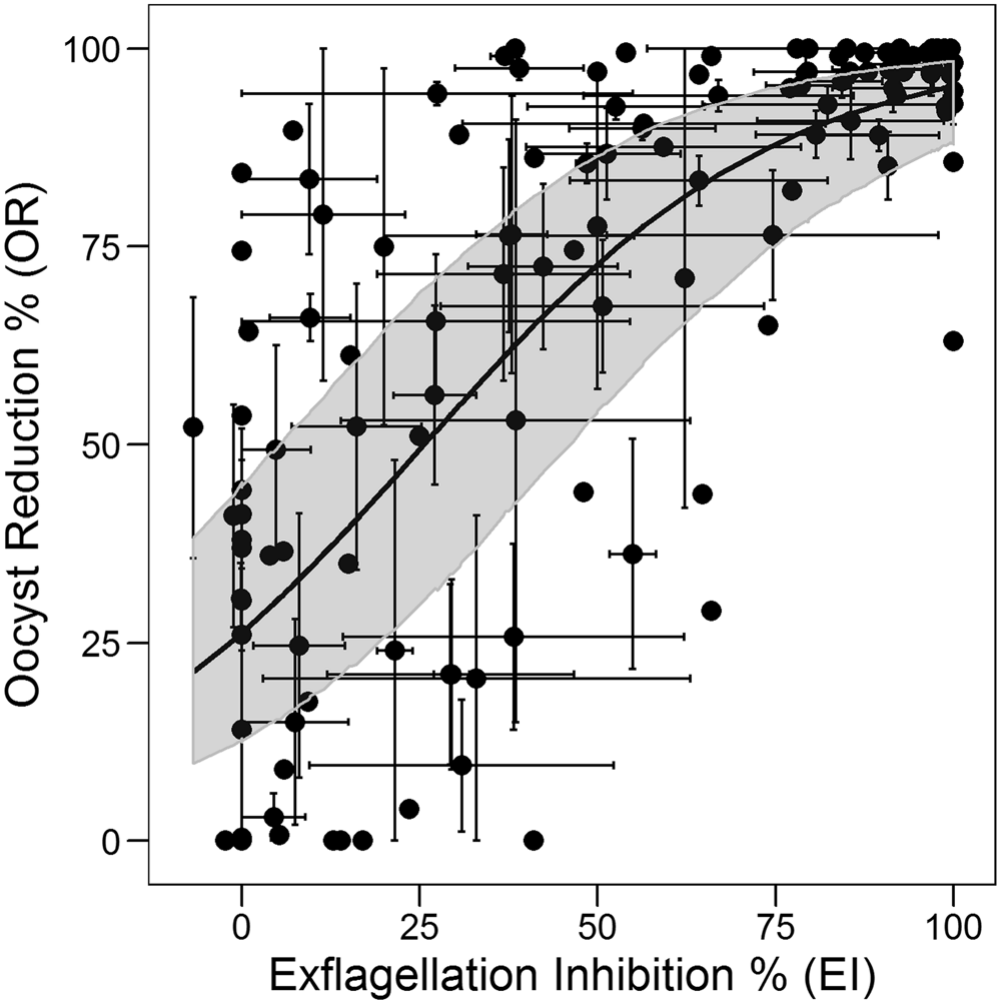
Relationship between EI and OR. Scatter plot of EI vs. OR (% of inhibition/reduction, see Eqs 1 and 2) for 40 compounds tested at different concentrations (total 131 data points and 14,882 fed mosquitoes). Each black dot represents an average measurement of one compound at one concentration, while each compound was screened either at a single concentration or up to eight different concentrations in both EIA and SMFA as described in Supplementary Table 1. Each point is the average of up to three repetitions of EIA and SMFA, with bars corresponding to standard errors. Best-fit is displayed as a black line (Eq. 4) together with the 95% confidence bands (dark grey shaded area around the curve) obtained through Monte Carlo simulations including both EI and OR errors.

In order to derive and test regression models, the 44 compounds described in Table 1 were randomly split into two groups: one group comprising 40 compounds with their corresponding 131 data points, was used to create a regression training set to derive the model; and another group of 4 compounds with their associated 17 data points was used as the validation set (Supplementary Table 1). The relationship between EI (% exflagellation inhibition, see Eq. 1) and OR (% oocyst reduction, see Eq. 2) of the 40 compounds of the training set was analyzed through regression and classification techniques and the resulting model was validated through internal cross-validation and external validation. Figure 1 represents a scatter plot of EI obtained from the compounds in the training set screened in EIA, versus OR obtained from the corresponding SMFA. Large variability was observed, due to assay complexity, but the data points show a clear positive association between the two variables, with a large concentration of data points at EI of ~100% and OR of ~100%. The data show a saturation effect where, for high EI values the OR value tends asymptotically to 100. It was therefore decided to fit the data to a 4-parameter logistic regression model with asymptotic values set to 0 and 100, respectively. The best fit equation is:

4 $$OR=\,\frac{100}{1+{10}^{0.017(24-EI)}}$$

with a Root Mean Square Error (RMSE) of 22.51%. The model provides the average OR expected in the mosquito when EI activity of a particular compound is known.

**Figure 2:**
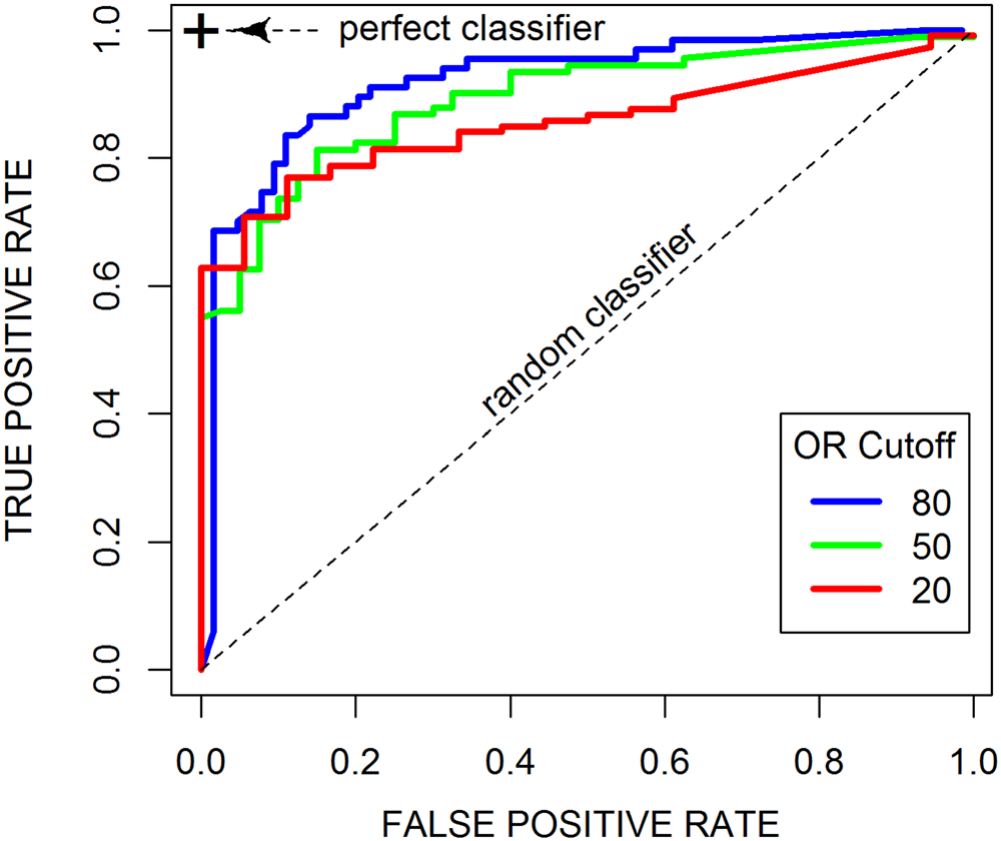
ROC curves of OR classification with EI. Empirical ROC curves for OR binary classification at moving cutoffs of EI. Curves are shown for binarization cutoffs for OR of 20 (red), 50 (green), and 80 (blue).

The ability of the EIA to predict and separate high from low OR values can be quantified by calculating the true positive and false positive rates for a given threshold of OR at different values of EI. In this way, several Receiver Operating Characteristic (ROC) curves can be derived at different thresholds of OR to assess the classification power of the EI parameter for the OR outcome in the mosquito. In these curves, at continuously varying thresholds of the classifier (EI) the true positive rate (in our case, of the compounds with an OR above a fixed threshold e.g. 20%, the fraction that show also an EI above the moving threshold) is plotted against the false positive rate (that is, of the compounds above the moving EI threshold, the fraction that are also below the OR fixed threshold). A perfect classifier corresponds to a curve that goes straight up in the Y axis to coordinate (0, 1) and then horizontally at y = 1, with an AUC = 1; a random classification yields a diagonal, with AUC = 0.5. ROC curves were generated for fixed thresholds of OR of 20%, 50% and 80% (Fig. 2), resulting in Area Under the Curve (AUC) values of; 0.92, 0.89 and 0.85, respectively. These high values indicate the capacity of the EIA to effectively separate high from low OR-valued samples.

From the data and the model, we can see that when EI = 0 we obtain an average OR of 24% indicating that even if some compounds demonstrate no exflagellation inhibition, they still could show some effect on oocyst reduction (there were still some compounds showing positive OR value). This is probably due to the nature of the data set used; compounds for SMFA were selected based on previous activity in other gametocytocidal assays thereby enriching for OR-active molecules. Regardless, the rate of false negatives is low, as reflected in the average value of 24%. On the other hand, the prediction at high EI values of 80 and more is very good and highly accurate, with no compounds in the high-EI, low-OR region (no false positives). This shows that molecules with an EI of 80% or more in the EIA most certainly will reduce the oocyst intensities by more than 80% in the mosquito.

To assess the predictive power of the procedure and type of model used, a leave-out-one cross-validation was performed, where each data point is removed, and then a model is derived with the rest of the points, which is then used to predict the former. This approach gave a RMSE of 22.93%, similar to that obtained in Eq. 4, indicating no overfitting.

**Figure 3:**
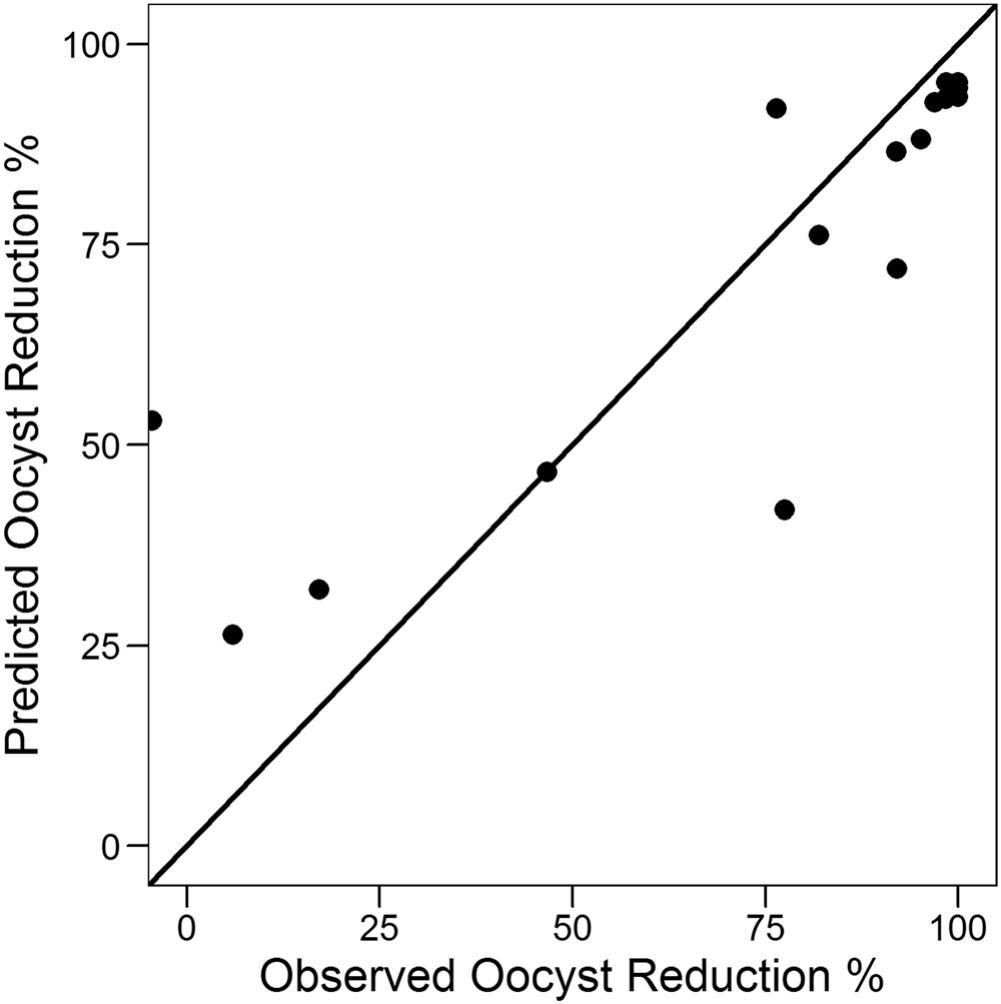
Prediction of OR. Experimental vs. predicted OR for external set (4 compounds, 17 data points and 1747 fed mosquitoes). Predictions were obtained using Eq. 4. Diagonal line is shown in black, corresponding to perfect prediction.

To externally validate the model, we used 4 compounds (validation set) tested at different concentrations (17 data points in total) for which the EI, OR and BIT had already been determined (described in Supplementary Table 1). These were evaluated in the same conditions as the training set (Fig. 1), and using Eq. 3 the observed EI was used to predict the OR. Figure 3 shows the observed versus the predicted OR for this external validation data set of 17 points, with an RMSE of 19.27%, again showing no overfitting and a reasonable prediction of OR. Once more, for high-EI compounds we see an accurate and reliable high OR prediction.

### Relationship between Oocyst Reduction (OR) and Block in Transmission (BIT)

**Figure 4:**
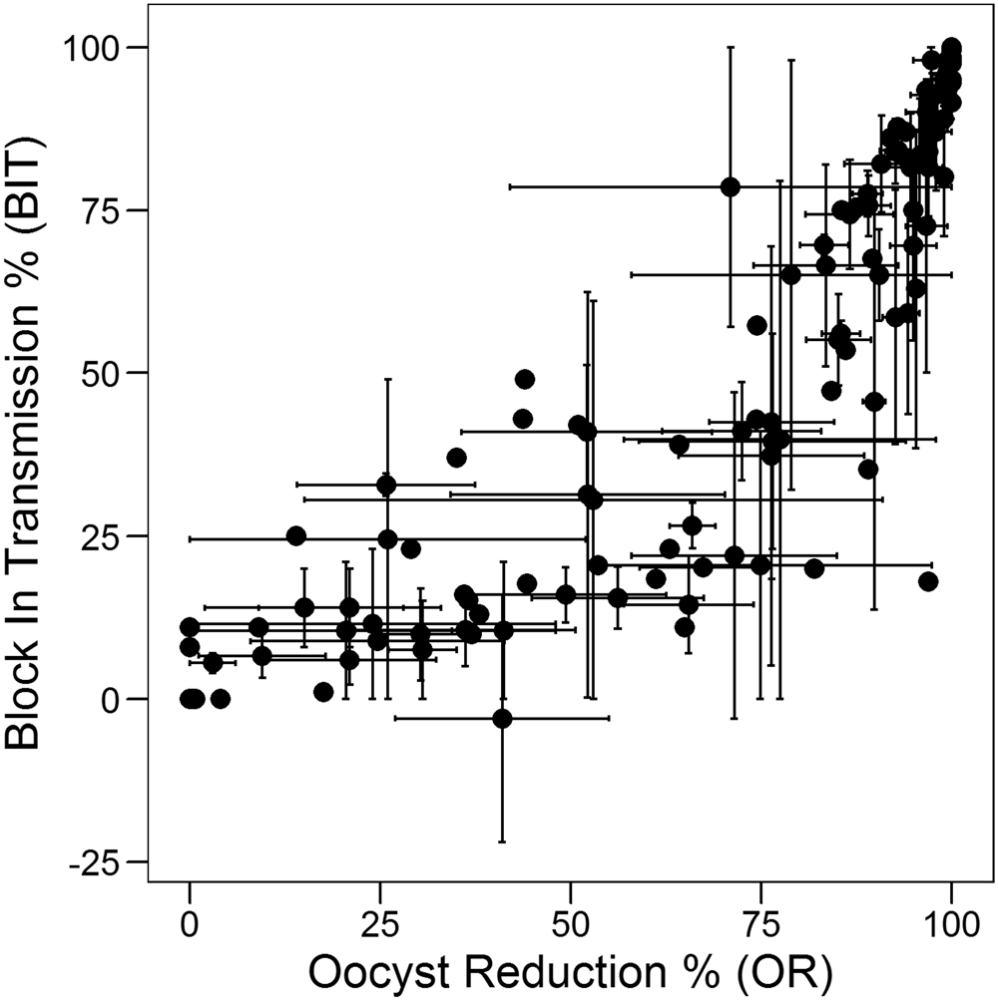
Relationship between OR and BIT. Scatter plot of OR vs. BIT for the 40 different compounds and concentrations as described in Fig. 1. Each point is the average of up to three repetitions of SMFA with a total of 14,882 fed mosquitoes, with error bars corresponding to standard errors.

OR and BIT are the control-normalized versions of reduction in oocyst mean intensity and prevalence, which are two parameters measured to characterize the effectiveness of a transmission blocking intervention. While the former is derived from the average number of oocysts per mosquito midgut (Eq. 1), the latter represents the proportion of mosquitoes having one or more oocysts (Eq. 2). Therefore, they provide two alternative aspects of the intervention that must be considered^9,19^. Uing SMFA data obtained from all 40 compounds used in the previous regression, we investigated the relationship between the OR and BIT, which is displayed as a scatter plot (Fig. 4). A nonlinear relationship between these two variables is observed. Similar behavior has been described by other authors, who have modeled it by means of a normal negative binomial distribution^20^ or more recently, by a zero-inflated negative binomial distribution^19^.

### Relationship between Exflagellation Inhibition (EI) and Block in Transmission (BIT)

**Figure 5:**
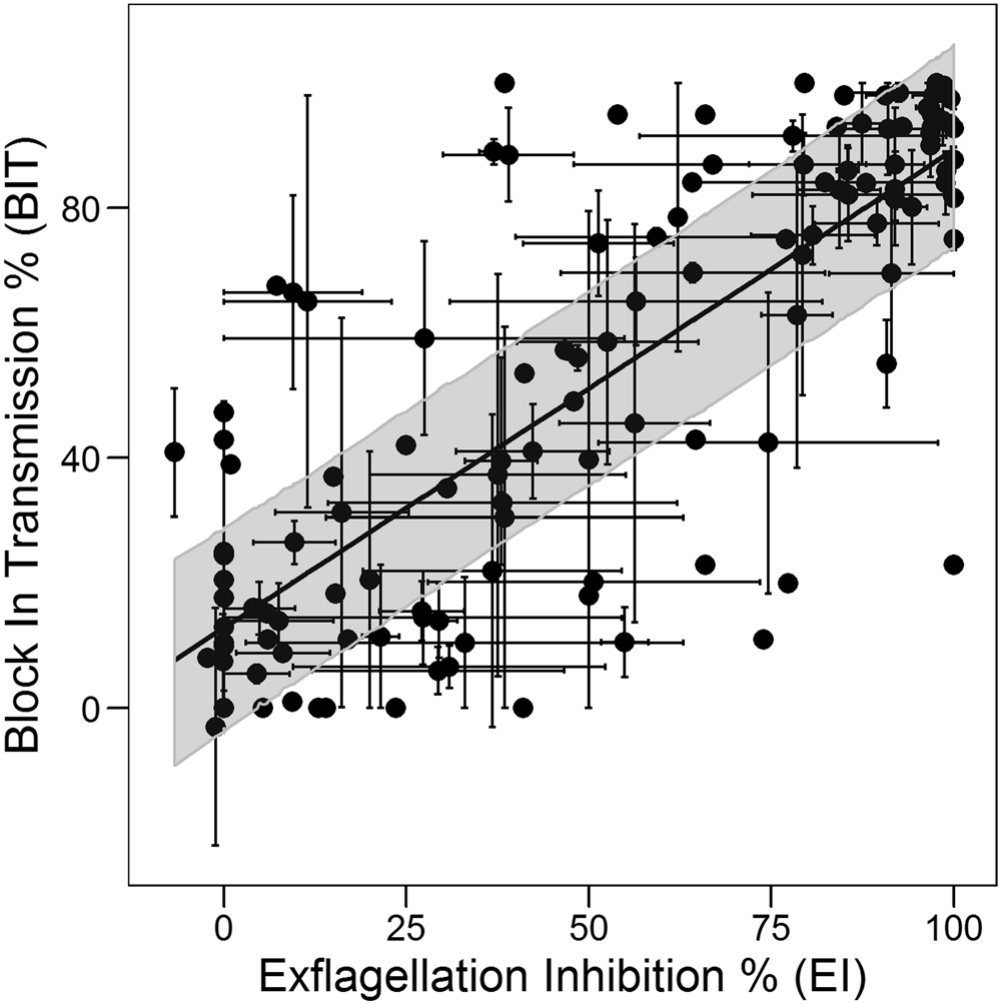
Relationship between EI and BIT. Scatter plot of EI vs. BIT for the compounds and concentrations as in Fig. 1. Each point is the average of up to three repetitions, with error bars corresponding to standard errors. Best-fit line is displayed as a black line (Eq. 5) together with the confidence bands (dark grey) obtained through Monte Carlo simulations including both EI and BIT errors.

Based on the positive correlation between the OR and BIT, as well as between EI and OR, we proceeded to establish the relation between the EI and BIT for the 40 different compounds from the same training set used above and the scatter plot of EI vs. BIT is shown in Fig. 5. Similar regression and classification techniques that were used to obtain Fig. 1, were also used here. The resulting model was validated with internal cross-validation and external validation set as described above.

In this case, no saturation occurred and therefore a straight line was fitted to the points, resulting in the following best-fit equation:

5 $$BIT=0.76EI+12.93$$

This corresponds to an RMSE of 21.07% (26.37% in leave-out-one cross-validation). The nonlinear relationship between EI and OR is compensated by the nonlinear relationship between OR and BIT, so that the resulting EI versus BIT relationship seems to be best described by a linear model.

**Figure 6:**
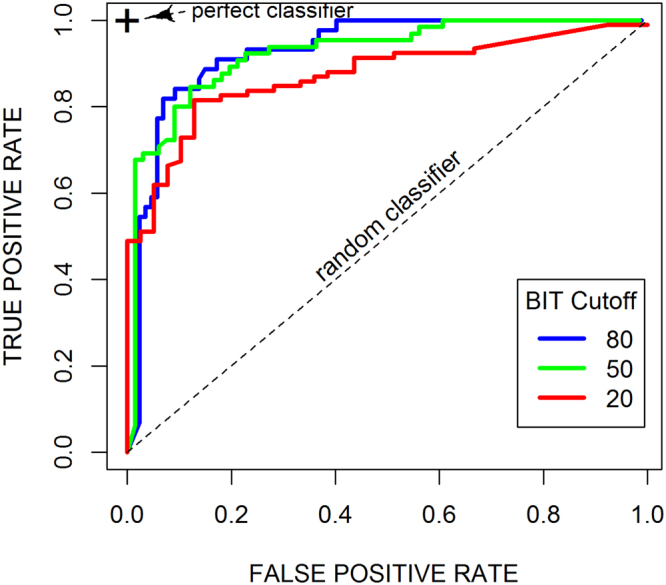
ROC curves of BIT classification with EI. Empirical ROC curves for BIT binary classification at moving cutoffs of EI. Curves are shown for binarization cutoffs for BIT of 20 (red), 50 (green), and 80 (blue).

Empirical ROC curves were similarly calculated for the EI vs. BIT relationship as shown in the Fig. 6, for thresholds of BIT of 20%, 50% and 80%. The resulting AUC are of 0.93, 0.92, and 0.87, respectively, which are similar values as those obtained for the EI vs. OR relationship. Thus, from this analysis we can conclude that the EIA separates well compounds with low BIT from those with high BIT, and that the EI can also be used as a predictor for BIT.

**Figure 7:**
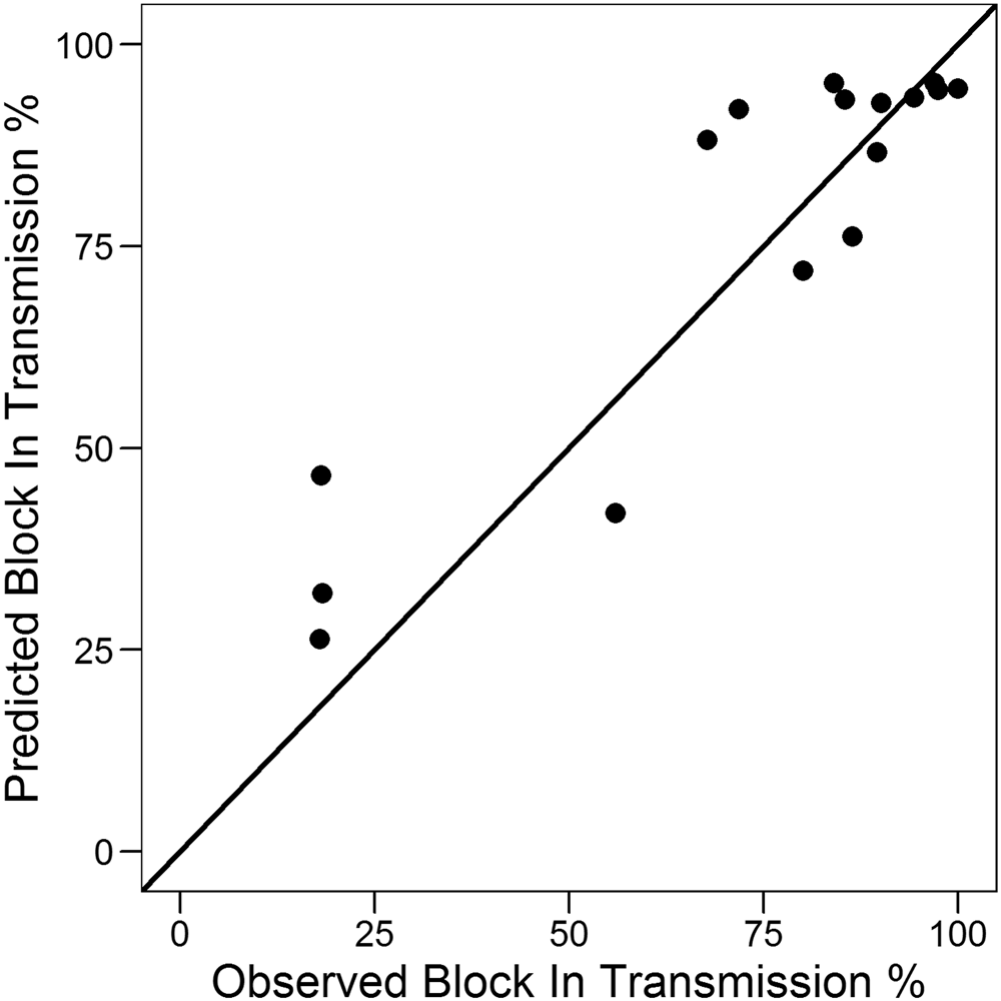
Prediction of BIT. Experimental vs predicted BIT for external set (4 compounds, 17 data points). Predictions were obtained with Eq. 5. Diagonal line is shown in black, corresponding to perfect prediction.

The model was validated with the external validation set (17 points) as described previously in Fig. 3 and also in Supplementary Table 1, giving an RMSE of 19.72% between the observed and the predicted BIT. The good predictive power of the linear model is depicted in Fig. 7.

### Relationship between Exflagellation Inhibition pIC50 and pIC50’s of Oocyst Reduction (OR) and Block in Transmission (BIT)

**Figure 8:**
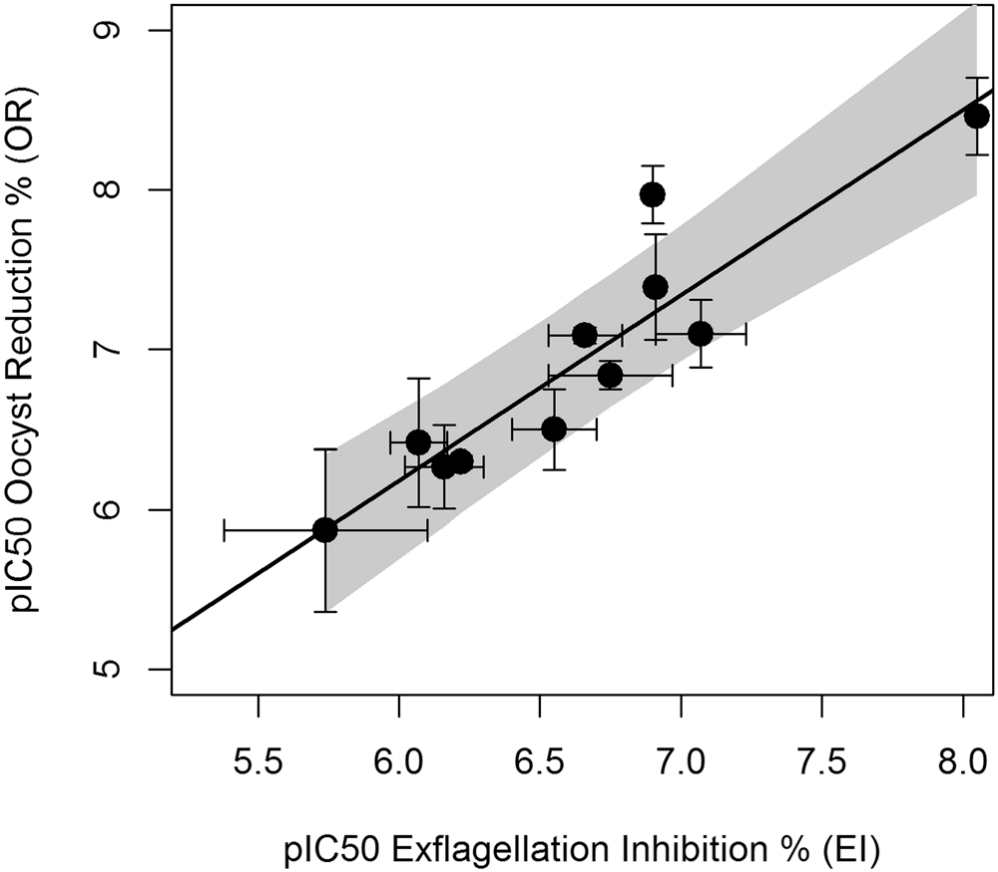
Relationship between EI pIC50 and OR pIC50. Scatter plot of EI pIC50 vs. OR pIC50 for 11 compounds with a total of 5761 fed mosquitoes. Each point is the mean of up to four EIA’s and SMFA’s, with error bars corresponding to the standard errors. Best-fit line (Eq. 6) is shown in black; 95% confidence bands including both pIC50-EI and pIC50-OR errors are included in dark grey.

We further wanted to establish if we could predict the IC50 values for OR and BIT using EI IC50 values. Using IC50 data from a set of 11 compounds (training set) we performed regression analsyis and the resulting models were validated using a set of four compounds (validation set) (Supplementary Table 2). EIA and SMFA was performed at up to 5 different compound concentrations for 11 different compounds and dose-response curves were obtained for EI, OR and BIT and IC50 determined. The corresponding pIC50’s (pIC50 = −log_10_ IC50) were estimated. Figure 8 shows the average pIC50 of EI versus the average pIC50 of OR for these compounds.

A significant linear relationship is observed; the best-fit linear regression model (black line in the Figure) corresponds to equation

6 $$pIC{50}_{OR}=1.16\ast pIC{50}_{EI}+0.75$$

with an r^2^ = 0.85, p < 0.05.

**Figure 9:**
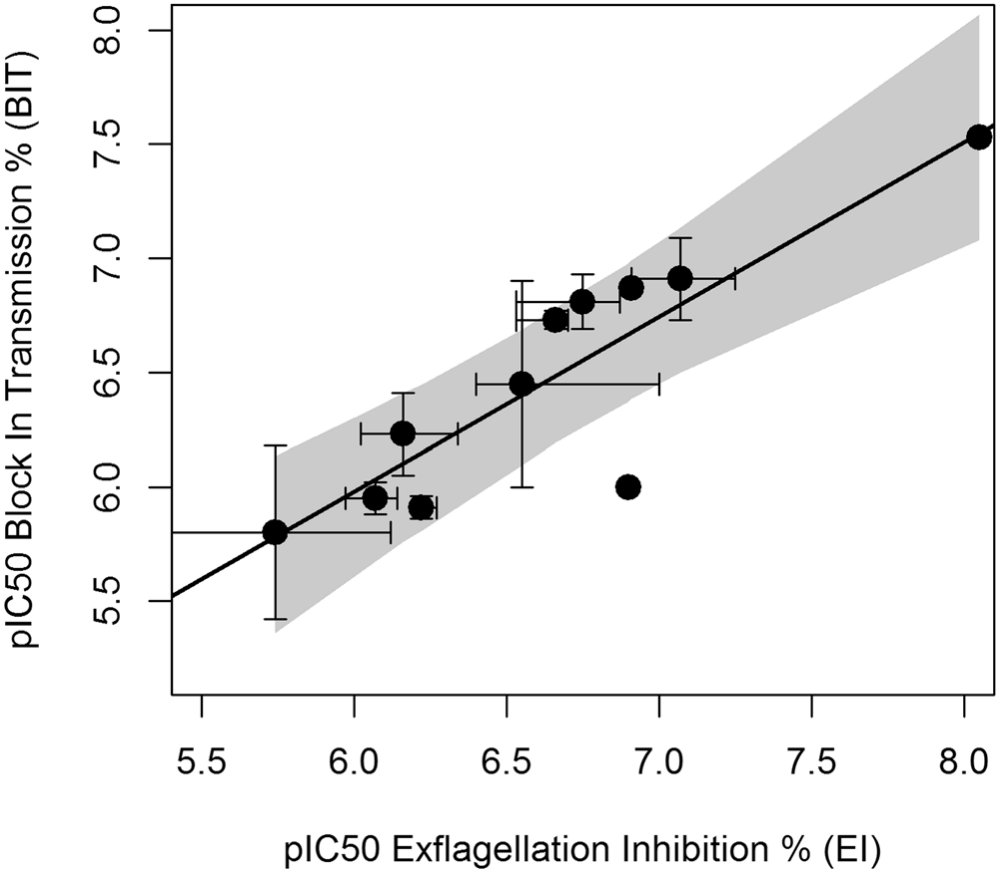
Relationship between EI pIC50 and BIT pIC50. Scatter plot of EI pIC50 vs. BIT pIC50 for 11 compounds. Each point is the mean of up to four measurements, with error bars corresponding to the standard errors. Best-fit line (Eq. 7) is shown in black; confidence bands including both EI and BIT pIC50 errors are included in dark grey.

This model was validated with an additional set of 4 molecules tested in the same conditions as in the training set in a total of 11 EIAs and SMFA’s with a total of 2929 fed mosquitoes (Supplementary Table 2). The actual pIC50 for EI was determined for these molecules, as well as the pIC50 for OR. Using Eq. 6, the pIC50 of OR was predicted and the results are described in Table 2. A reasonable prediction (RMSE = 38.1%, maximum difference of 0.6 pIC50 units) of the OR pIC50 from the EI pIC50 was observed. Similarly, as with pIC50’s of EI versus OR, a significant linear relationship was observed between the EI and BIT pIC50’s, as shown in Fig. 9.In this case, the equation for the best-fit line is:

7 $$pIC{50}_{BIT}=0.76pIC{50}_{EI}+1.4$$

with an r^2^ = 0.76, p < 0.05.

**Table 2:** Prediction of OR pIC50 from EI pIC50.

Compound Number	EI pIC50	Actual OR pIC50	Predicted OR pIC50
43	7.1	7.3	7.5
37	6.1	6.6	6.3
41	6.5	7.4	6.8
42	7.1	7.7	7.4

EI and OR pIC50’s were determined from EIA and SMFA respectively for four different compounds. Using the EI pIC50 values in Eq. 6 the OR pIC50 was predicted and compared with actual values obtained.

To validate this model, the BIT pIC50s were also determined for the external set of 4 compounds (Supplementary Table 2). Table 3 shows the predicted and actual BIT pIC50’s, together with the EI pIC50. A decent agreement (RMSE = 23.9%, maximum difference of 0.3 pIC50 units) is observed between predicted and actual values.

**Table 3:** Prediction of BIT pIC50 from EI pIC50.

Compound Number	EI pIC50	Actual BIT pIC50	Predicted BIT pIC50
43	7.1	7.1	6.8
37	6.1	6.3	6
41	6.5	6.5	6.4
42	7.1	7	6.8

EI and BIT pIC50’s were determined from EIA and SMFA respectively for same set of compounds used in Table 2. Using EI pIC50 values in Eq. 7 the BITpIC50 was predicted and compared with the actual values obtained.

These two equations are in agreement with the previous observed associations in the single-concentration data (Figs 1 and 4), and confirm the ability of the EIA to predict OR or BIT IC50 of compounds. The slopes in eqs 6 and 7 above are close to 1, although in the case of BIT a trend is observed in the pIC50 of EI to under predict the pIC50’s of BIT.

## Discussion

SMFA outcome is interdependent on gametocyte infectivity and mosquito susceptibility to *P*. *falciparum* infection, as well as the number of mosquitoes in each treatment. A useful parameter for determining infectivity of mature gametocytes to mosquitoes after the long *in vitro* culturing process relies on the ability of male gametocytes to form functional motile male gametes. In our laboratory, we not only used this assay to determine culture viability but also used it to study the inhibitory effect of compounds on male gamete formation. The same drug-treated gametocytes were used both, in EIA and for preparation of bloodmeal for mosquito feeds in SMFA. This is extremely advantageous as we could analyze the outcomes of the same parasites in two different assays enabling a direct correlation between the outcomes of an *in vitro* assay based on inhibition of male gamete formation, and the final output in the mosquito in terms of reduction in oocyst intensity and prevalence. We employed a data set derived from the analysis of a total of 40 compounds assayed either at a single or multiple concentrations (131 data points) using a total of 14,882 mosquitoes. This allowed us to estimate regression models for both EI vs. OR (in the form of a four-parameter logistic equation) and EI vs. BIT (in the form of a simple linear regression model) at a single concentration. The models were internally cross-validated by leave-one-out cross-validation, and externally validated with a validation set, showing good predictive powers in both cases with an error similar to that of the training set. The observed trend seems to be general and not dependent on chemical family. In addition, simple linear regression models were derived to predict pIC50’s of OR and BIT from pIC50’s of EI, which gave reasonable predictions when used with an external validation set of compounds. Both, these models derived from either the single concentration or pIC50 data, show the efficacy of the EIA in predicting SMFA outcomes, and can be used in the prioritization of compounds to be tested in SMFAs.

The positive outcome of parasite infection in the mosquito depends on the successful fertilization of male and female gametes in the mosquito midgut to form a zygote which transforms to a motile ookinete that crosses the peritrophic matrix and the midgut epithelium and subsequently differentiates into an oocyst at the basal lamina. Compounds that disrupt the formation of male and/or female gamete and which prevent the downstream ability to form zygote will interfere with the ability to form oocysts. Although the readout in our study was based on the inhibition of male exflagellation, one cannot ignore the fact that compounds could also have an effect on female gamete formation, although to the best of our knowledge in our screens we have not obtained compounds with selective activity only against females. Besides, male gametocytes have been reported to be more susceptible to anti-malarial’s compared to female gametocytes^7^. Our data strongly suggest that the effect of compounds on male gamete formation, without relying on the effect on the female gamete formation, can by itself predict the outcome in the mosquito. Nevertheless, in Figs 1 and 4, we see that some compounds screened at concentrations showing no EI activity did show some degree of OR and BIT. This is reflected in the derived models, where the intercepts are 24 and 12.9, respectively for Eqs 3 and 4. This could be attributed to the fact that the compounds could be affecting female and not male gametocyte and therefore interfering with zygote formation. Besides, high protein/RBC binding properties of some compounds could prevent removal in the washing step during preparation of bloodmeal for SMFA, enabling carryover into the mosquito during feeding. This could influence stages of parasite development in the mosquito thereby interfering with oocyst formation which was our final readout of SMFA. Out of the 44 compounds used in this study we found 6 compounds with zero exflagellation inhibition and two with some amount of enhanced exflagellation showing varying degrees of inhibition of oocyst intensity and prevalence (Supplementary Table 1). This corresponds to a total of 14 out of the total 148 averaged points used in our regression analysis and overall contributes to a low rate of false negatives. In addition, given the inherent noise of these complex biological systems, the assay could have some degree of false positives. For instance, in the prediction of OR in the external validation set (Fig. 3) one data point (out of 17) was predicted to display >50% OR while the experimental observation was near zero.

Given the complexity of SMFA, it becomes imperative to define stringent selection criteria for progression of compounds into the mosquito. In our platform at GSK, only compounds with pre-established gametocytocidal activity in any one of the several gametocyte-based *in vitro* assays were selected for screening in SMFA. Although this selection criterion is beneficial in filtering out compounds which do not have gametocytocidal activity, it can be challenging to decide the exact concentration at which we can screen for TrB activity in mosquitoes. In previous studies by our group^21,22^, we used the IC90 values obtained from the gametocytocidal ATP based assay^15^ and the female gamete activation assay (FGAA)^16^ as a starting concentration for screening for TrBP of TCAM hits in SMFA. Besides providing essential information about new chemical structures and scaffolds to serve as starting points for drug discovery programs, results from this initial single point SMFA also help give an idea about the range of concentrations for performing a more robust full dose-response mosquito feeding assay but these are not always informative. It would be advantageous to have simple and easy to use predictive tools derived from *in vitro* assays (as presented here) which could estimate TrBP in the mosquito thereby pre-empting the need to perform several SMFAs in order to determine concentration range and TrB activity.

Using the data from Figs 1 and 4, we can differentiate compounds with TrB into three separate categories based on their behavior in EIA and SMFA. Compounds with EI of 75% or more can be categorized as high-probability TrB compounds, as they mainly gave ORs or BITs above 75% (especially in the case of OR, given the saturating behavior observed); those with EI between 25% and 75%, as medium-probability TrB, as they result in an enriched set of TrB compounds, but with some proportion of inactive compounds. This is enhanced more when BIT is used as an outcome than OR. And finally, those with less than 25% EI as low-probability TrB compounds with a predominance of compounds inactive in SMFA. Using a fixed concentration of 1 µM as a starting point for evaluation we propose a critical path for progression of compounds into SMFA. This starting concentration of 1uM will serve as a threshold concentration to enable selection of compounds with transmission blocking activity in the nanomolar range. The first step will be to perform EIA at 1 µM and ascertain whether the compound can be categorized as a high-, medium- or low-probability TrB. A full dose response in EIA starting at a concentration of 1 µM will have to be performed using compounds with medium to high probability TrB followed by determination of the EI pIC50. Using Eqs 6 and 7, the EI pIC50 will be used to predict the OR and BIT IC50’s in SMFA. Based on these predicted values, we can decide on the range and number of concentrations at which to perform SMFA to determine the actual OR and BIT of the compound in the mosquito. However, compounds which are categorized as having poor TrBP based on the EI values, cannot be written off as having no TrBP considering that even at zero EI, we do see OR and BIT in mosquitoes for some compounds. Nevertheless, if the EI for these compounds at a fixed concentration of 1 µM is very low, there is a low probability that these will show high OR and BIT in the SMFA. Depending on the stage of development of these compounds (late lead or pre-clinical candidate) such compounds can be progressed into SMFA at a single concentration of 1 µM to confirm activity in the mosquito, thus obviating the need to perform tedious dose dependent SMFA’s at several concentrations with large numbers of mosquitoes.

In conclusion, this study is unique in that it uses identical parasites in two different assays and therefore shows the direct correlation between results from an *in vitro* male gamete formation assay and mosquito feeding assay. Using simple regression models, we have demonstrated the ability of efficiently using an *in vitro* generated outcome to predict TrB ability of compounds in complex *in vivo* biological systems. Further, using models obtained from these analyses we proposed a simple pathway for progression of molecules in SMFA thereby contributing to efficient use of mosquitoes and slots for screening molecules with TrB in mosquitoes.

## Materials and Methods

### Mosquito colony

Mosquito rearing facilities are located at Tres Cantos Medicines Development Campus at GSK (Madrid, Spain). *Anopheles stephensi* colony was established in 2013 from eggs kindly provided by Michael Delves and Mark Tunncliffe from Imperial College, London. Mosquitoes were maintained in climate controlled chambers (Panasonic MLR 352-H) at a temperature of 26.5 ± 1 °C, 14 L:10D photoperiod and a relative humidity of 75 ± 5%, with *ad libitum* access to 10% glucose/water solution + 1% Karo® syrup.

### Gametocyte production

Gametocyte cultures were generated from *P*. *falciparum* NF54 (BEI Resources, MRA-1000) and 3D7 (BEI Resources, MRA-1001). Asexual’s were maintained at a maximum of 1% total parasitemia and 5% hematocrit in RPMI1640 with L-glutamine (15.87 g/L), 25 mM HEPES, 10 mM Glucose, 20 mM Bicarbonate, 5 mM Hypoxanthine. Media was made complete with 10% of human A^+^ serum (Interstate Blood Bank). Human erythrocytes Type A+ obtained from Biobank of Castilla y Leon, BST and Centro de Transfusiones de Madrid. Gametocyte culture protocol was adapted as previously described^22^. Gametocyte induction was initiated at 0.5% parasitemia (>70% rings) and 4% hematocrit in RMPI1640 with 30 mM bicarbonate and 5 mM hypoxanthine at 50 mL final volume in T75 flasks. Media was made complete with 5% human serum A^+^ and 0.5% albumax (5% Albumax II-Sigma from 20X stock solution). Cultures were maintained for up to 20 days with daily media change and without addition of fresh RBC in a gassed incubator at 37 °C, 5% CO2, 5% O2, 90% N2. At day 13 to 15 post-induction, media was replaced with a serum only gametocyte treatment media (same as asexual culture media described above). Cultures were monitored daily after day 13 using Giemsa stained smears for Stage V gametocytemia, male and female gametocyte ratio and by performing exflagellation assay for viability.

### Compounds

Data in this study was generated using results from a total of 44 different compounds belonging to different chemical classes and were either leads, late leads, pre-clinical candidates or in clinical stages of development (Table 1) and are referred to in the figures and tables in the Results section as compound 1, 2, 3 etc. Compounds were prepared fresh as a stock solution of 10 mM in 100% of DMSO and added to mature gametocytes as described below. These were screened either at a single concentration or in a full dose at four or five different concentrations.

### Drug treatment

*In vitro* cultures used for EIA and SMFA had gametocytemia between 1 to 3% Stage V, male to female ratio of not less than 1:2 and exflagellations of more than 20 centers/field at 100X total magnification. Gametocyte cultures (5 mL) were incubated for 24 hours with the required concentration of compound in the same final concentration of DMSO (0.1%) and an aliquot (100 µL) of these cultures was used in the EIA described in the method below. For some compounds, exposure was performed for 48 hours. If gametocytes were treated for 48 hours, at 24 hours post drug-exposure a fixed volume (3 mL) of spent media was removed and fresh media was re-added accompanied by compound replenishment to obtain the required final concentration. Untreated gametocytes with the same final concentration of DMSO (0.1%) as in treated gametocytes were processed in parallel.

### Exflagellation inhibition assay

Exflagellation Inhibition Assay (EIA) was performed prior to all SMFA’s and was initially performed by manual counting of microscopic exflagellation centers and later by capturing movement of exflagellating centers over time by video microscopy.

For microscopic enumeration of exflagellation centers we adapted a method described by Ghosh *et al*.^23^,. 100 µL of mature gametocyte culture (day 14-day 20 post-induction) was centrifuged at 1800 rpm for 30 seconds. Packed cells were resuspended in 15 µL of pre-warmed (37 °C) ookinete medium (RPMI medium with 25 mM HEPES, 50 mg/L hypoxanthine, 2 g/L sodium bicarbonate, 100 μM xanthurenic acid, 20% human serum). and then introduced in a chamber of a FastRead 102 (Immune Systems Inc.) disposable hemocytometer slide which was placed on a horizontal surface at room temperature (RT) to allow cells to homogeneously settle. The slide was incubated at RT and the time noted as time zero (T_0_) and then observed under a light microscope at 40X objective for counting of exflagellation centers between minute 15 and 20, in a total of 25 fields. The average number of exflagellation centers per field was determined. For semi-automated enumeration, movement of exflagellating centers was recorded over time by video microscopy using the Leica DM 4000B microscope, fitted with a Leica DFC 310FX camera at 10X magnification and then quantified by a semi-automated method using a modification of a method described by Ruecker *et al*.^8^ and as previously reported^22^. A series of 8 videos of 2 seconds each are captured at random locations on the slide with an exposure of 1 ms between 18 to 22 minutes after incubation. Each video is analyzed as a series of matrices to create a single image using Image J application^24^ and number of clusters were analyzed with the Cell Profiler Application^25^.

### SMFA

SMFA was performed as previously described. On the day of the feed, drug treated mature *P*. *falciparum* gametocyte cultures were centrifuged at 2500 × g for 3 minutes at 37 °C and the supernatant was removed along with any of the compound which was added in the treatment process described above. The pellet was diluted 1:1 with 100% packed cell volume of fresh human RBC’s Type A + and finally formulated as artificial mosquito blood meals at 50% hematocrit with pre-warmed human serum. All steps were performed at 37 °C. Prepared blood meals were fed in duplicate to overnight starved 4–6 days old female *An*. *stephensi* mosquitoes (40 mosquitoes/ cup) for the duration of 30–40 minutes via Parafilm membrane attached to glass feeders connected to a 37 °C circulating water bath. Each SMFA is run with internal duplicates (2 cups for each compound concentration). Fed mosquitoes were maintained in an incubator at 26 ± 1 °C, 14 L:10D photoperiod and a relative humidity of 75 ± 5%. 7–8 days post-feeding mosquitoes with fully developed ovaries (to select out feds from unfeds) were dissected for midguts (Leica, M80) which were incubated in 0.2% mercurochrome solution in D/W for 10–15 minutes. Total number of oocysts in individual midguts were counted using a light microscope (Leica, DM2000) using a 10X Objective (100X magnification). Both, infection prevalence (percentage of mosquitoes with one or more oocyst) and mean oocyst intensity of infection was defined in each treatment. The oocyst load is compared between the treated and un-treated control groups. A minimum of two independent SMFA replicates were performed for each compound evaluated except if mentioned otherwise (Supplementary Table 1).

### IC50 determination

The inhibitory effect of the compound on each of the parameters observed (exflagellation centers per field, mean oocyst intensity, prevalence of infection) were normalized to the respective DMSO treated control and % inhibition (y) calculated. Percentage inhibition data obtained from each individual parameter was fitted to a four-parameter logistic equation with variable slope using GraphPad Prism 6.07 for which the compound concentrations (x) were first transformed to respective log_10_ values. The data were fitted using these transformed values of x without defining constraints. The IC50 values were calculated from the anti-log of the x value corresponding to the 50% inhibition obtained by interpolating the XY coordinate table obtained in GraphPad Prism. pIC50’s were calculated from the IC50 as described in Results.

### Statistical analysis

All the statistical analyses and graphs were generated with the software R, version 3.4.1. For the linear regressions, the stats package was used. For the nonlinear regressions, the nls package was used. The pROC package was employed for the ROC analyses, and the ggplot2 and base packages for generating the plots. The 95% confidence bands of the regression models (grey bands in the figures), were obtained through Monte Carlo simulations that included both the error in EI and SMFA parameters (OR and BIT). To internally validate the models, leave-out-one cross validations were performed. The regression models were applied to external validation sets to ascertain their predictive power in data not used to train the models (external validation). As a measure of error the Root Mean Squared Error (RMSE) was used.

### Ethics statement

All methods in this study were carried out in accordance with GlaxoSmithKline guidelines and regulations. Human biological samples used in the study have been obtained in accordance with all relevant laws including, the Human Tissue Act 2004 and the Medical Research Council (MRC) Guidelines entitled “Human Tissue and Biological Samples for use in Research” regarding the collection, use and transport of human tissue. Human red blood cells used in the study were obtained from Biobank of Castilla y Leon, BST and Centro de Transfusiones de Madrid and the relevant ethics committee approvals have been obtained from Autonoma University of Madrid (CEI-45-890) to enable the use these samples from human subject volunteers or other donors. All use of human biological samples in this study was in accord with the terms of the informed consents given by the sample donors. All experiments for use of mice were ethically reviewed and approved by the GlaxoSmithKline Diseases of the Developing World (DDW) Group Ethical Committee on Animal Research and were conducted according to Spanish legislation, European Directive 2010/63/EU and GlaxoSmithKline policy on the Care, Welfare and Treatment of Laboratory animals.

## Electronic supplementary material

Supplementary Information

Table S1.

Table S2.
