# Supplementary material for: Predicting transmission blocking potential of anti-malarial compounds in the Mosquito Feeding Assay using Plasmodium falciparum Male Gamete Inhibition Assay
Source: Sci Rep. 2018 May 17;8:7764. doi: 10.1038/s41598-018-26125-w (PMC5958111; doi:10.1038/s41598-018-26125-w)
Supplement: Supplementary file 1 — Supplementary Information [file 41598_2018_26125_MOESM1_ESM.pdf]

Predicting transmission blocking potential of anti-malarial compounds in the Mosquito Feeding Assay using *Plasmodium falciparum* Male Gamete Inhibition Assay

Authors:

Gonzalo Colmenarejo[1,4§], Sonia Lozano-Arias[1], Carolina González-Cortés[1,5§], David Calvo[1], Juliana Sanchez-Garcia[2], Jesús-Luís Presa Matilla[1], Didier Leroy[3], Janneth Rodrigues[1*]

[1]Diseases of the Developing World (DDW), GlaxoSmithKline, Severo Ochoa 2, Tres Cantos 28760, Madrid, Spain

[2]*In vivo* Science & Delivery (IVSD), GlaxoSmithKline, Severo Ochoa 2, Tres Cantos 28760, Madrid, Spain

[3]Medicines for Malaria Venture, Route de Pré-Bois 20, 1215 Geneva 15, Switzerland

[4]Biostatistics and Bioinformatics Unit, IMDEA Food Institute, CEI UAM + CSIC, Ctra Cantoblanco 8, 28049 Madrid, Spain

[5]Complejo Asistencial Universitario de León. Altos de Nava s/n, 24071 León, Spain.

[§] Current address

*Corresponding Author

janneth-fatima-indira.x.rodrigues@gsk.com

Supplementary Information

Table S1. SMFA and EIA single-concentration information for the complete list of 44 compounds assayed for this work (training and validation sets).

Table S2. SMFA and EIA pIC50 information for the complete list of 15 compounds (training and validation sets) assayed for this work.
